# Using a Disentangled Neural Network to Objectively Assess the Outcomes of Midfacial Surgery in Syndromic Craniosynostosis

**DOI:** 10.1097/PRS.0000000000011686

**Published:** 2024-08-20

**Authors:** Alexander J. Rickart, Simone Foti, Lara S. van de Lande, Connor Wagner, Silvia Schievano, Noor ul Owase Jeelani, Matthew J. Clarkson, Juling Ong, Jordan W. Swanson, Scott P. Bartlett, Jesse A. Taylor, David J. Dunaway

**Affiliations:** London, United Kingdom; Rotterdam, the Netherlands; and Philadelphia, PA; From the ^1^UCL Great Ormond Street Institute of Child Health and Craniofacial Unit, Great Ormond Street Hospital for Children; 2 Department of Computing, Imperial College London; 3 Department of Oral and Maxillofacial Surgery, Erasmus MC; 4 Division of Plastic, Reconstructive and Oral Surgery, Children’s Hospital of Philadelphia; 5 Centre for Medical Image Computing; 6 Wellcome/EPSRC Centre for Interventional and Surgical Sciences, University College London.

## Abstract

**Background:**

Advancements in artificial intelligence and the development of shape models that quantify normal head shape and facial morphology provide frameworks by which the outcomes of craniofacial surgery can be compared. In this work, the authors demonstrate the use of the swap disentangled variational autoencoder to assess changes after midfacial surgery objectively.

**Methods:**

The model is trained on a data set of 1405 3-dimensional meshes of healthy individuals and syndromic patients, which was augmented using a technique based on spectral interpolation. Patients with a diagnosis of Apert or Crouzon syndrome who had undergone sub- or transcranial midfacial procedures using rigid external distraction had their results interpreted using this model as the point of comparison.

**Results:**

A total of 56 patients met the inclusion criteria: 20 with Apert syndrome and 36 with Crouzon syndrome. By using linear discriminant analysis to project the high-dimensional vectors derived by swap disentangled variational autoencoder onto a 2-dimensional space, the shape properties of Apert syndrome and Crouzon syndrome can be visualized in relation to the healthy population. In this way, the authors were able to show how surgery elicits global shape changes in each patient. To assess the regional movements achieved during surgery, the authors used a novel metric derived from the Mahalanobis distance to quantify movements through the latent space.

**Conclusions:**

Objective outcome evaluation, which encourages in-depth analysis and enhances decision-making, is essential for the progression of surgical practice. The authors demonstrate how artificial intelligence has the ability to improve our understanding of surgery and its effect on craniofacial morphology.

Recent advances in artificial intelligence and deep learning have led to their integration into our everyday lives, driving advancements in personalized recommendations, facilitating autonomous vehicle operations, and enhancing health care diagnostics.^1^ In keeping with this, the field of geometric morphometrics has shown great promise in leveraging these tools to make steps toward the deep phenotyping of craniofacial syndromes.^2–4^ However, the diagnosis of these conditions remains multimodal, combining clinical evaluation, imaging, neurocognitive assessment, and advances in genetic sequencing.^5,6^ As such, the true translational benefit of these approaches lies in improving the granularity with which we can understand the presenting phenotype and aid in shaping patient management on a personalized level.^7^

A longstanding challenge of craniofacial surgery is how to approach the objective assessment of aesthetic outcomes. Subjective assessment tools have understandably shown variable results, and patient-reported outcome measures have difficulty teasing apart the intertwined psychosocial implications of appearance.^8–13^ In contrast, the development of shape models that quantify normal head shape and facial morphology provide a potential frame of reference by which outcomes can be compared.^14–17^ Building on initial work analyzing the shape properties of craniofacial syndromes that focused on anthropometric methods, more recent developments use deep learning applied to 2-dimensional (2D) images in the instance of DeepGestalt or in 3 dimensions when considering the work of Hallgrímsson et al.,^3^ O’Sullivan et al.,^4^ and others.^2,9,13^ Technical developments have led to improved performance and diagnostic accuracy, and, in some cases, improved interpretability.^18–22^

Mesh autoencoders have shown patients with syndromic craniosynostosis to tightly cluster by diagnosis when visualized using t-distributed stochastic neighbor embedding.^4^ Furthering the insights of this work, the swap-disentangled variational autoencoder (SD-VAE) has improved our understanding of Apert, Crouzon, and Muenke syndromes by considering not only the global morphology, but also the influence of each anatomic subunit on the overall phenotype.^20^ As we can quantify how each region of the face correlates with the healthy population, it will also be possible to measure the influence of surgery in exerting changes at these regions.

We demonstrate the use of SD-VAE to assess changes objectively following both subcranial and transcranial procedures undertaken on patients with Apert or Crouzon syndrome. Quantification and comparison of morphology with the healthy population is undertaken considering overall appearance as well as the shape changes influenced at the local level.

## PATIENTS AND METHODS

### Data Sources

Ethical approval was gained from the joint research and development office at the Great Ormond Street Hospital for Children (GOSH), London; the UK Research Ethics Committee (UK REC 15/LO/0386); and the institutional review board at the Children’s Hospital of Philadelphia (CHOP), Pennsylvania (approval no. 12-009276). Informed consent for participation was obtained from the patients and their parents or legal guardians.

Access to the implementation of mesh preprocessing, SD-VAE, and its application to craniofacial surgery is freely available at github.com/simofoti.

The SD-VAE model used in this study was pretrained on head meshes of patients with Apert (*n* = 39), Crouzon (*n* = 53), or Muenke (*n* = 11) syndrome, as well as healthy participants (*n* = 250).^4,15,20^ The database was augmented using spectral interpolation to return 1405 syndromic meshes.^23,24^ Age range of the participants was 1 day to 20 years, with a male to female ratio of 55:45. Information on race and ethnicity was not available. Further details on this control population are available in the work by O’Sullivan and colleagues.^4^

Review of the craniofacial databases at both centers allowed identification of new patients with combined clinical and genetic diagnoses of Apert and Crouzon syndrome, and who had undergone sub- or transcranial midfacial procedures using rigid external distraction between 2005 and 2022. This included monobloc, midfacial bipartition, monobloc with differential Le Fort II advancement, and Le Fort III osteotomies. For the purposes of this study, we did not consider patients with Muenke syndrome further, owing to a paucity of postoperative 3-dimensional (3D) surgical data. Patients were included if high-resolution computed tomography was available at the preoperative stage and within 18 months following the completion of distraction. Computed tomography scans with ≤1-mm slice thickness were required, and those where soft-tissue thresholding revealed distortion or artifact were excluded. The included patients were then processed to be analyzed with the pretrained SD-VAE.

### Data Processing

Using the Materialise suite of software (Materialise NV), DICOM files were converted to manifold 3D surface meshes of the face, head, and neck using thresholding and isolation techniques. The resulting meshes were placed in dense point correspondence with the template used to build our original model,^4,17^ first using nonrigid iterative closest point registration guided by standardized landmarking, followed by Gaussian processes (Scalismo; University of Basel) that preferentially affected global head shape over complex regional features, such as the ears and nose.^4,25–27^ Processing the meshes in this way ensures that they have analogous topology, with an identical number of vertices interconnected through consistent triangulation. This consistency means that each corresponding vertex has the same semantic meaning across different meshes, allowing for accurate calculation of movements across the many thousands of vertices.

### Manifold Visualization

SD-VAE learns to map patient meshes into vectors laying on a latent manifold, which is a low-dimensional space that compactly captures complex relationships in the patient meshes. The latent manifold can be further projected in a 2D space for visualization and to understand these mesh relationships.

We used linear discriminant analysis (LDA) to project the 75-dimensional latent vectors obtained by processing patient meshes with SD-VAE onto a 2D space in which the global shape properties of Apert and Crouzon syndromes, as well as the healthy population, are grouped into different distributions. With this providing perspective, we were then able to superimpose preoperative and postoperative data points in this latent representation to demonstrate how craniofacial surgery influences the shape properties of each patient. In the same way, it was also possible to project into a 2D space the 5-dimensional subsets that represent the different anatomic subunits because of the latent disentanglement capabilities of SD-VAE. This again provides context on how each region changes with respect to the norm after surgery.

It is worth clarifying that a shape space and the latent space are 2 different ways of analyzing changes in craniofacial structures. Shape space directly represents detailed meshes of the head. In contrast, latent space is an abstract, compact statistical form. When projected into 2D space, it simplifies the complexities, making interpretation easier. This dual perspective allows us to understand and delineate changes in more detail.

### Objective Assessment of Surgical Outcomes

The latent vectors and their attribute-specific subsets were not only visualized with an LDA-based 2D projective transformation, but also classified with quadratic discriminant analysis (QDA) models. QDA is a generalization of LDA that assumes vectors of different classes to follow Gaussian distributions, each parametrized by a different covariance matrix. As in Foti and colleagues’ work,^28^ these QDA models can be used in conjunction with SD-VAE to automate diagnosis. In addition, the covariances estimated with QDA were used to compute more meaningful distances in the latent space. In particular, said $\Sigma {}_{H}^{r}$ the covariance of the healthy distribution in the head region, the Mahalanobis distance was computed between 2 regional latent vectors, $z_{1}^{r}$ and $z_{2}^{r}$, as:


$$\begin{array}{l} d_{M}\left(z_{1}^{r},z_{2}^{r}\right)=\sqrt{\left(z_{1}^{r}-z_{2}^{r}\right)\Sigma {}_{H}^{r}{\left(z_{1}^{r}-z_{2}^{r}\right)}^{T}}. \end{array}$$

**Fig. 1.**
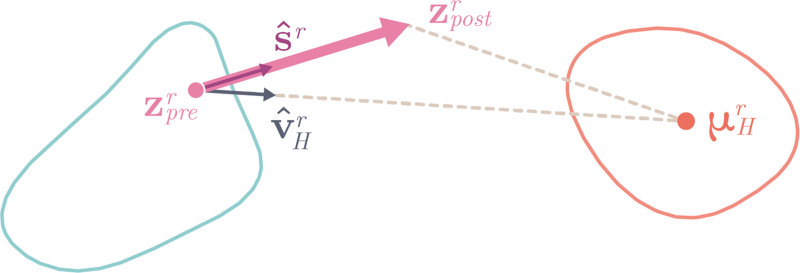
A 2D representation of the shape properties of the orbits of a patient with Apert syndrome (*pink dot*) projected over the Apert and healthy distributions (*blue* and *red contours*, respectively). The *pink arrow* represents how the orbits of a single patient changed in shape after surgery, from $z_{pre}^{r}toz_{post}^{r}$. The *gray arrow* ${\hat{v}}_{H}^{r}$ is the versor indicating the direction between the preoperative shape and the mean of the healthy distribution. The metric of surgical outcome is designed to consider how significant the region-by-region movements are in relation to this proposed ideal trajectory, the proximity of the postoperative properties to the healthy ones, and actual preoperative to postoperative changes.

While Euclidean distances between latent vectors have no meaningful scale, the Mahalanobis distance is measured in terms of standard deviations from the healthy distribution.^29^ For instance, if $d_{M}\left(z_{1}^{r},z_{2}^{r}\right)=1$, $z_{1}^{r}$ and $z_{2}^{r}$ are at 1 SD from each other. Leveraging this distance, we defined a regional metric to assess surgical outcomes as follows:


$$\begin{array}{l} m^{r}=\frac{d_{M}\left(z_{pre}^{r},z_{post}^{r}\right)}{d_{M}\left(z_{post}^{r},\mu_{H}^{r}\right)}‹{\hat{s}}^{r},{\hat{v}}_{H}^{r}›, \end{array}$$

where $z_{pre}^{r}$ and $z_{post}^{r}$ are the latent subsets of the head region *r* obtained from the preoperative and postoperative meshes of a patient’s head, respectively; $\mu_{H}^{r}$ is the mean of the region-specific latent healthy distribution; ‹⋅,⋅› is an inner product; ${\hat{s}}^{r}$ is the versor indicating the direction of the vector representing the surgical movement of region *r*; and ${\hat{v}}_{H}^{r}$ is the versor indicating the trajectory from the preoperative representation to the center of the healthy distribution. These variables can be visualized in Figure 1, where they are projected into a 2D space for visualization purposes. Intuitively, high $m^{r}$ values are associated with procedures that significantly altered the latent representation in the correct direction and that determined a postoperative result that is close to the center of the healthy distribution. The magnitude of the alteration is computed by $d_{M}\left(z_{pre}^{r},z_{post}^{r}\right)$, the direction is evaluated by computing the inner product, and the distance to the center is determined by $d_{M}\left(z_{post}^{r},\mu_{H}^{r}\right)$.

## RESULTS

A total of 56 patients met our strict inclusion criteria: 20 with Apert syndrome and 36 with Crouzon syndrome (Table 1). A total of 38 had undergone previous surgery on the posterior vault: posterior vault distraction osteogenesis (CHOP), spring-assisted posterior vault expansion (GOSH), or conventional posterior cranial vault remodeling (both centers). Fifteen patients had undergone previous fronto-orbital remodeling at a younger age. Further details including the timing of imaging relative to surgery are summarized in Table 1.

**Table 1.** Characteristics by Diagnosis and Procedure Type

Characteristics	Apert Syndrome				Crouzon Syndrome	
	Bipartition	Monobloc	Monobloc + Le Fort II	Le Fort III	Monobloc	Le Fort III
No. of patients	11	3	3	3	28	8
Sex, no. male/female	5/6	1/2	1/2	1/2	13/15	4/4
Patient age, median (range), yr	13.5 (2.3–23.2)	5.5 (4.8–6.3)	8.1 (5.5–11.7)	7.1 (5.4–8.4)	11.3 (0.9–18.8)	9.7 (4.6–19.8)
Days between preoperative imaging and surgery, median (range)	190 (25–566)	34 (16–76)	39 (23–48)	35 (17–82)	126.5 (4–272)	232.5 (58–479)
Days between postoperative imaging and surgery, median (range)	292.5 (56–479)	139 (126–141)	112 (98–172)	141 (98–226)	151.5 (6–256)	136 (72–205)
Previous posterior vault surgery, no.	7	3	2	2	21	3
Previous fronto-orbital advancement, no.	0	2	2	3	2	6

### Classification

**Fig. 2.**
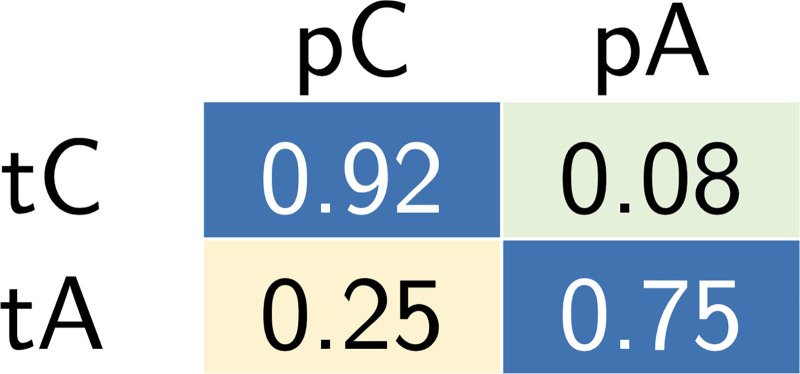
Confusion matrix reporting the classification results on the preoperative out-of-distribution data using the global pretrained QDA model. The rows of the matrix correspond to the labels true Crouzon (*tC*) and true Apert (*tA*), and the columns to the labels predicted Crouzon (*pC*) and predicted Apert (*pA*).

By computing the latent representation of the preoperative patients with SD-VAE, and using them as inputs to the pretrained QDAs, classification was achieved with a precision rate of 92% when identifying patients with Crouzon syndrome and 75% for those with Apert syndrome. The results, which should be interpreted in the context of being out of distribution, are summarized in the confusion matrix in Figure 2.

### Global Movements

**Fig. 3.**
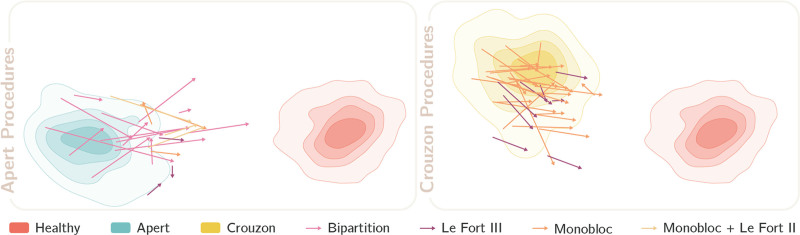
Manifold visualization of the global shape attributes of patients with Apert or Crouzon syndrome, and how these are influenced by a range of midfacial and frontofacial surgical procedures. As in Figure 1, each patient is characterized by an *arrow*, with the tail representing the preoperative latent of the patient and the head of the arrow equating to the postoperative outcome. The color of the arrow indicates the surgical procedure. Arrows are projected in a 2D space where patients can be compared against the typical distributions of both the healthy and syndromic populations. In light of this, we expect arrows to originate from the syndromic distribution and move toward the healthy distribution, with longer arrows equating to larger morphologic changes.

By using LDA to project the high-dimensional vectors derived by SD-VAE onto a 2D space, the shape properties of Apert and Crouzon syndromes can be visualized in relation to the healthy population. Using this as a backdrop, Figure 3 shows how surgery elicits global shape changes in each patient. Patients who have a presurgical starting point further away from the center of their syndromic representation and closer to the healthy distribution have had previous surgery or represent a milder phenotype. Each arrow represents a surgical procedure, where a longer arrow equates to a larger change in overall morphology. These arrows consistently demonstrate the positive movements toward the healthy population brought about by surgery. However, when considering the distance from the mean healthy distribution to our superimposed surgical outcomes, there appears to be scope for improvement.

### Regional Movements

**Fig. 4.**
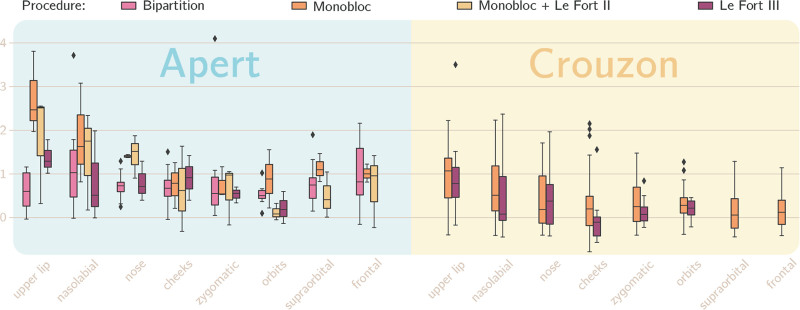
Boxplot demonstrating the regional changes brought about by surgery, as quantified using our objective metric delineated in the Patients and Methods section. A larger value indicates a more significant shape change in the direction toward the healthy distribution.

To quantify the regional movements achieved during surgery, we used the metric outlined previously, whereby a larger value indicates a more significant shape change in the direction toward the healthy distribution (Fig. 4). Observing the Crouzon cohort in the first instance, both monobloc and Le Fort III show similarities in the magnitude to which they exert changes in the morphology of the midface. As expected, there is no change in the supraorbital or frontal regions for those undergoing subcranial Le Fort III.

In comparison, the effects on regional anatomy are more varied in the Apert cohort, where a wider range of procedures is represented. The strength of multipiece osteotomies is shown by the ability of the monobloc with differential Le Fort II to significantly change the projection of the central midface to create a more balanced facial appearance. The upper lip and nasolabial regions also demonstrate pronounced changes in the monobloc group. In comparison, the changes in these regions are more modest in the midfacial bipartition group, which may be a reflection of the initial adverse movements at the maxillary level. The remaining anatomic subunits demonstrate less variation among the 4 included procedures.

**Fig. 5.**
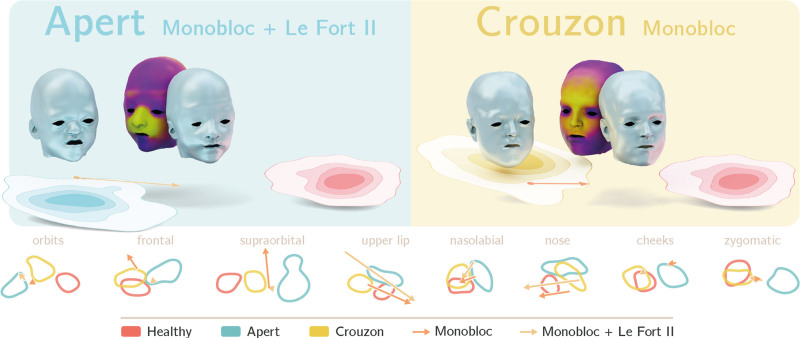
Patient-specific examples of how SD-VAE can be used to assess global and regional shape changes achieved by surgery. (*Left*) An 11-year-old girl with Apert syndrome who was not previously operated on. (*Right*) A 15-year-old girl with Crouzon syndrome who had previously undergone spring-assisted posterior vault expansion. (*Above*) Renders of preoperative and postoperative facial morphology are projected above the manifold visualization of their global movements toward the healthy distribution. Adjacent to the postoperative render is a heatmap quantifying the changes, where *yellow* represents movements greater than 10 mm. (*Below*) Changes across each anatomic subunit are represented with respect to the Apert, Crouzon, and healthy populations.

Using a combination of the global and regional assessments applied to individual cases, it is also possible to demonstrate how using SD-VAE can provide detailed objective feedback on surgical outcomes (Fig. 5).

## DISCUSSION

Our application of SD-VAE demonstrates how artificial intelligence can be used to improve our understanding of craniofacial surgery. By disentangling the regional anatomy, it is possible to improve the detail with which we can appraise surgical outcomes, while retaining global context.

At first glance, the global shape changes after surgery, as demonstrated in Figure 3, may appear underwhelming. However, the model has a diagnostic sensitivity much greater than the human eye. It also places value in regions of the head and neck that may not immediately be associated with the syndromic phenotype and indeed on those not altered by surgery. Similarly, LDA promotes class differentiation and so, on manifold visualization, broadens the distance between the healthy and syndromic populations. This goes some way to explain the slight disconnect between the research and clinical picture, where both subcranial and frontofacial surgery are powerful tools for addressing the facial dysmorphism seen in Apert and Crouzon syndromes. Instead, when considering the global picture, it is more useful to compare surgery across a patient population, or, as we have done here, across a range of procedures.

The manifold visualization of the preoperative patient meshes move slightly further from the mean syndromic population if they have had previous fronto-orbtial surgery, as is the case for the majority of the patients with Crouzon syndrome undergoing Le Fort III advancement (Fig. 3). However, when comparing the surgical end points with patients undergoing monobloc advancement, the global shape outcomes for both surgical procedures are similar. Given that this is the case, the argument may circle back toward the risks and benefits of an approach focusing on early fronto-orbital remodeling followed by a Le Fort III procedure later in life versus a single-stage frontofacial operation. Considering the safety profile of transcranial surgery in modern craniofacial surgery, consideration should be given to minimizing the number of surgical interventions to achieve an optimal functional and aesthetic outcome.^30–34^

The development of a metric by which regional shape changes can be compared holds promise for future evaluation of surgical outcomes. We have demonstrated the ability of both multipiece and monobloc procedures to address the midfacial biconcavity seen in Apert syndrome (Fig. 4). Both conventional monobloc advancement and monobloc with differential Le Fort II produce the most significant changes in the central midface. This adds further weight to the evidence that rigid external distraction elicits a beneficial plastic deformity within the frontofacial monobloc segment by exerting a central pull on the region and improving projection.^35^ In comparison, the reduced impact of facial bipartition at the upper lip and nasolabial regions is likely a manifestation of the early unfavorable movement at the maxillary level. Instigated by the medial rotation of the orbits around a midfacial pivot, this typically results in a midline diastema and a posterior crossbite, but can also provide a vertical discrepancy between the 2 maxillary segments if an asymmetric orbital movement is required. This settles with time and the aid of orthodontic management, but here may account for the lower metric in these regions.^36^ However, this demonstrates the ability of this measure to detect and quantify recognized disadvantages to different surgical approaches.

Three-dimensional representation of each anatomic subunit provides the option for patient-specific assessment in both the preoperative and postoperative phases. As shown in Figure 5, appraisal of surgical outcomes on a case-by-case basis provides a detailed understanding of where a case went well, and where it could be improved. In the cases shown, of particular interest are the shape changes at the nose and upper lip. The fact that the arrows for these regions move in the direction of the healthy population and then subsequently past it are in keeping with the desired overcorrection. Again, this lends further credence to the ability of SD-VAE to assess the effects of surgery.

Although this work establishes the potential for deep learning methodologies to be integrated into clinical practice and aid in the assessment of craniofacial surgery, there are certain caveats. When considering rare craniofacial syndromes, one of the key limitations is the volume of data available. This is exacerbated by the nature of soft tissue, where imaging with compressed or distorted anatomy must be excluded, narrowing an already small pool of patients. Advances in 3D photogrammetry will likely help to overcome this, as repeating the imaging has less consequence, but obtaining high-quality images in infants remains a challenge owing to movement artifacts and hair interference.^37^

A philosophical and technical consideration is the use of mean healthy populations as a proposed target for measuring movements. In practice, craniofacial surgery has dual functional and aesthetic components, aiming to reshape the head toward a more normal appearance within the constraints of human anatomy. This is particularly relevant when considering the timing of midfacial surgery, often performed before a child begins high school, with the goal of reducing the physiologic burden associated with living with a craniofacial syndrome. Both GOSH and CHOP have published extensively on the outcomes and complications of the relevant cohorts. Given the previously reported positive functional results, normalizing head shape aligns intuitively with these primary functional outcomes.^30,33–35,38–42^

Leading on from this, a future avenue for investigation would be to assess how geodesic latent trajectories may differ from the path we have taken here. In this instance, the optimal surgical pathway toward normal would not follow straight lines (like the one between $z_{pre}^{r}$ and $\mu_{H}^{r}$ in Fig. 1), but instead follow a curved pathway that prioritizes certain regions in the latent space.^43,44^ A methodology of this nature would necessitate a greater volume of data in the first instance.

## CONCLUSIONS

Objective outcome evaluation, which encourages in-depth analysis and enhances decision-making, is essential for the progression of surgical practice. We have demonstrated how artificial intelligence has the ability to improve our understanding of surgery and its effect on craniofacial morphology. Future work in this field will look to explore the correlation between objective and patient-reported outcomes, use geodesic latent trajectories, and track the changes in morphology as a patient grows from infancy to adulthood.

## DISCLOSURE

Dr. Swanson is a consultant for KLS Martin, LP, and Synthes, LP. Dr. Taylor is a cofounder of Ostiio, LLC. The remaining authors have no financial interests to report, and the authors have no conflicts of interest to declare.

## ACKNOWLEDGMENTS

This work was funded by the Great Ormond Street Hospital for Children Charity (grant 12SG15), the Engineering and Physical Sciences Research Council (EP/N02124X/1), the European Research Council (ERC-2017-StG-757923), the Wellcome Trust (203145Z/16/Z), and the National Institute for Health Research Biomedical Research Centre Funding Scheme. The views expressed in this publication are those of the authors and not necessarily those of the National Health Service, the National Institute for Health Research, or the Department of Health. The funders had no role in study design, collection, analysis, or interpretation of the data; decision to publish; or preparation of the manuscript.
